# European validation of an image-derived AI-based short-term risk model for individualized breast cancer screening—a nested case-control study

**DOI:** 10.1016/j.lanepe.2023.100798

**Published:** 2023-12-06

**Authors:** Mikael Eriksson, Marta Román, Axel Gräwingholt, Xavier Castells, Andrea Nitrosi, Pierpaolo Pattacini, Sylvia Heywang-Köbrunner, Paolo G. Rossi

**Affiliations:** a Department of Medical Epidemiology and Biostatistics, Karolinska Institutet, Stockholm, Sweden; b Department of Public Health and Primary Care, University of Cambridge, UK; c IMIM (Hospital del Mar Medical Research Institute), Barcelona, Spain; d Mammographiescreening Paderborn, Paderborn, Germany; e Azienda Unitá Sanitaria Locale-IRCCS di Reggio Emilia, Reggia Emilia, Italy; f Referenzzentrum Mammographie Munich, Brustdiagnostik München and FFB gGmbH, Munich, Germany

**Keywords:** Breast cancer, Risk prediction, Artificial intelligence, Validation, Mammography screening, Europe

## Abstract

**Background:**

Image-derived artificial intelligence (AI)-based risk models for breast cancer have shown high discriminatory performances compared with clinical risk models based on family history and lifestyle factors. However, little is known about their generalizability across European screening settings. We therefore investigated the discriminatory performances of an AI-based risk model in European screening settings.

**Methods:**

Using four European screening populations in three countries (Italy, Spain, Germany) screened between 2009 and 2020 for women aged 45–69, we performed a nested case-control study to assess the predictive performance of an AI-based risk model. In total, 739 women with incident breast cancers were included together with 7812 controls matched on year of study-entry. Mammographic features (density, microcalcifications, masses, left-right breast asymmetries of these features) were extracted using AI from negative digital mammograms at study-entry. Two-year absolute risks of breast cancer were predicted and assessed after two years of follow-up. Adjusted risk stratification performance metrics were reported per clinical guidelines.

**Findings:**

The overall adjusted Area Under the receiver operating characteristic Curve (aAUC) of the AI risk model was 0.72 (95% CI 0.70–0.75) for breast cancers developed in four screening populations. In the 6.2% [529/8551] of women at high risk using the National Institute of Health and Care Excellence (NICE) guidelines thresholds, cancers were more likely diagnosed after 2 years follow-up, risk-ratio (RR) 6.7 (95% CI 5.6–8.0), compared with the 69% [5907/8551] of women classified at general risk by the model. Similar risk-ratios were observed across levels of mammographic density.

**Interpretation:**

The AI risk model showed generalizable discriminatory performances across European populations and, predicted ∼30% of clinically relevant stage 2 and higher breast cancers in ∼6% of high-risk women who were sent home with a negative mammogram. Similar results were seen in women with fatty and dense breasts.

**Funding:**

10.13039/501100004359Swedish Research Council.

**Research in context**

**Evidence before this study**

Risk prediction of breast cancer using artificial intelligence showed promising results and has a potential for improving mammography screening outcomes. The performance of predictive models is known to potentially be affected by differences in screening routines and screening populations. Nevertheless, few studies have evaluated the generalizability of the predictive performances of such risk tools across multiple screening populations. We searched PubMed for search string: “breast cancer” AND (“risk prediction” OR “risk assessment”) AND “validation” AND (“artificial intelligence” OR “deep learning”) and found several AI-based risk models analyzing mammograms to identify mammographic features beyond breast density to predict risk of breast cancer in the general screening population. Studies reported predictive performances on 5-year risk, 2-year risk of breast cancer, and risk of interval cancer that occurs between screens. Discriminatory performances were reported to exceed an AUC of 0.7 and were found to be significantly higher than AUCs reported using traditional familial/lifestyle-based risk models with AUC performances below 0.7. We identified 2 external validation studies of AI-based risk-prediction tools using at least one large screening population. In both studies the tools showed similar performance in external and internal validation cohorts. However, we did not identify any study reporting the generalizability of the predictive performances across several European screening populations.

**Added value of this study**

Our study confirms that the AI-based risk model generalizes across investigated European screening populations with AUCs consistently 0.7 or higher. Our results also indicate that the AI-based risk assessment predicts later stage breast cancers as high risk among women who currently are sent at home with a negative mammogram.

**Implications of all the available evidence**

Risk assessment using an AI-based risk model demonstrates a mature technology designed for risk-stratified screening and, could enable pragmatic clinical trials aimed at exploring the integration of risk-based screening strategies into European screening programs.

## Introduction

After the introduction of mammography screening, women have benefited from a reduction in breast cancer related death due to the detection of breast cancer at an earlier stage.^1^ Recent estimations approximate a 20–40% reduction in deaths compared with women not participating in screening.^2^^,^^3^ However, in women screened biennially approximately 25% of breast cancers are still diagnosed after a negative screen but before the next scheduled screen.^4^ In addition, 25–40% of breast cancers are diagnosed at stage 2 or higher.^5^ Tumor stage and whether the tumor was screen detected or appeared between two screens are strong prognostic markers of breast cancer related mortality.^6^

The addition of risk assessment to the screening protocol has been proposed to improve screening further by identifying women who need additional examinations after a negative screening due to their high risk of interval cancer before the next screen or a late-stage breast cancer at the next screen.^7^

In the United States, hospitals are reimbursed for performing additional examinations in women who have dense breasts or a high risk of breast cancer due to familial risk factors.^8^ In Europe, most countries have organized national programs for breast cancer screening, screening guidelines do not currently suggest risk assessment in the general screening population,^9^ but trials are investigating risk stratified screening.^10^^,^^11^

Several clinical risk tools have been developed based on lifestyle factors and family history for improving screening outcomes and, in more recently, also image-based risk models.^12^^,^^13^ Promising results have been reported based on such newer risk models; however external validation is still sparse for judging the clinical feasibility of such models.^14^

For this reason, we performed an external validation of a previously developed image-derived artificial intelligence (AI)E-based risk model, which was designed to predict risk of breast cancer in the short-term, for its ability to predict women who return with a breast cancer before or at the next scheduled screen in two years after a negative screen. We used the ProFound AI Risk (iCAD Inc., Nashua, NH) model^15^^,^^16^ in a European setting including four screening populations in Italy, Spain, and Germany. The model is available for clinical use in the U.S. and Europe. We estimated the overall discriminatory performance and risk classification of the model.

## Methods

### Study population

In this pooled analysis of four cohort studies, women aged 45–69 were included who underwent mammographic screening in four screening populations. One in Italy, one in Spain, and two in Germany between 2009 and 2020, Supplementary Table S1. The screening populations are detailed in Supplementary Method S1.^17^, ^18^, ^19^, ^20^ From each screening population, we included incident breast cancers with a digital mammogram at study-entry and a diagnosis before or at the next screening round. Cancers were included from 3 months to 2 years and 3 months (90–820 days) after study-entry to account for lead time to diagnosis after breast cancer detection in screening. Our cancer outcome was defined based on register linkage after the end of study. Women with a personal history of breast cancer were excluded from the study. We performed a nested case control study in each population. For each population, controls were randomly selected from the underlying screening cohort and frequency-matched on the year of mammography to cases at study-entry. A total of 739 breast cancers and 7812 controls were included in the validation study. In the RETomo trial (Italy), 180 breast cancers and 1899 controls, at Hospital Del Mar (Spain) 158 breast cancers and 1550 controls, from München Süd (Germany) 232 breast cancer and 3124 controls, and from Paderborn (Germany) 169 breast cancers and 1239 controls were included. The study protocol is available as Appendix A.

In RETomo, women in age 45–49 had annual screens as part of the screening program. In the trial, all women had digital mammography at study baseline. At the next scheduled screen, where cancer outcome was assessed, half of the women were randomized into having digital mammography, arm 1, and the remaining half had also digital breast tomosynthesis (DBT), arm 2. This was done as part of the original randomized controlled trial (RCT) with the same name.^17^, ^18^, ^19^, ^20^ For our current study we had a different aim, where we studied risk assessment of breast cancer based on the prior mammograms and, reported risk of breast cancer at study baseline for the two outcome groups arm 1 and 2. For the Paderborn study population, we had no access to interval cancers.

The article followed the Strengthening the Reporting of Observational Studies in Epidemiology (STROBE) guidelines for cohort studies.^21^

This re-analysis study was approved for RETomo by Ethics Committee of Italy (2021/0040435, 24/03/2021), for Hospital Del Mar by Ethics Committee of Hospital del Mar Medical Research Institute (IMIM) (2021/9736, 9/June/2021), for München Süd in Germany, Bavaria, no ethics committee approval was required, since completely anonymized data and images were provided for the study, and for Paderborn by the ethics committee in Germany (2020-760-f-S). The requirement to obtain informed consent was waived.

### Mammographic risk factors and age at study-entry

Full-field digital mammographic (FFDM) images were obtained from the left and right breasts (mediolateral oblique (MLO) and cranio-caudal (CC) views). The mammograms were used to extract AI-based mammographic features (density, microcalcifications, masses, and left-right breast asymmetries of these features) using the ProFound AI Risk Model 1 tool (iCAD, Nashua, NH) and the STRATUS mammographic density tool as previously described.^15^^,^^22^ A detailed description is available in Supplementary Method S2.^15^^,^^22^ The risk tool is designed to identify women who may benefit from supplemental screening or a shorter screening interval due to high risk of breast cancer before or at the next scheduled screen.

Two-year absolute risks of breast cancer were calculated for each woman based on mammographic risk factors, age at study-entry, national statistics of breast cancer incidence rate, and competing mortality risk.^15^^,^^23^^,^^24^

### Breast cancers during follow-up

Breast cancer occurrence and tumor stage were retrieved from medical records using hospital-specific personal identification numbers for each population. Tumor stage was defined using the American Joint Committee on Cancer (AJCC) classification.^25^

### Statistical analysis

Descriptive statistics were used to summarize the characteristics of the study participants at study-entry. The time to breast cancer diagnosis after a negative mammogram was reported as a frequency distribution. The absolute 2-year risk was estimated at study-entry for each woman. All analyses were adjusted for age at study-entry, year of mammogram, and mammography machine vendor. Adjusted Area Under the receiver operating characteristics Curve (aAUC) estimated the discriminatory performance of the model for each population and across populations after adjustment.^26^ A detailed description is available in Supplementary Method S3.^26^^,^^27^ The 95% confidence intervals of the AUC point estimates were estimated using 1000 bootstraps. The Receiver Operating Characteristic (ROC) curve of the model sensitivities and specificities at different operating points were also reported after adjustment.^26^ Risk classification was performed using the European National Institute of Health and Care Excellence (NICE) and the U.S. Preventive Services Task Force (USPSTF) guidelines thresholds on absolute risks,^8^^,^^28^ where women were classified into high-, moderate-, and general risk groups. For NICE, we used the absolute risk cut-off value 0.6% to distinguish between general and moderate risk and 1.6% to distinguish between moderate and high risk. The corresponding thresholds were 0.24% and 1.2% for USPSTF. The cut-offs were based on the NICE recommendations for 10-year risks, where 3% is used to distinguish the low and moderate risk groups and 8% to distinguish the moderate and high-risk groups. As we predicted 2-year risks in our study, we approximated the 2-year risk cut-offs by dividing the 10-year cut-offs by 5. The cut-offs for the USPSTF were based on the 5-year absolute risks of 0.6% (the average risk of a 40-year-old woman) to distinguish the low and moderate risk groups and 3% to distinguish the moderate and high-risk groups. We adapted the cut-offs to 2-year risks by dividing the numbers by 2.5.^15^^,^^16^^,^^23^^,^^29^

In a sensitivity analysis, the risk classification was restricted to women of age 50 and above. Absolute risks were reported for cases and controls in density plots and, the numbers of cases and controls in each risk category were reported. Absolute risks were summarized as means for each risk group. Risk ratios were estimated by contrasting cases and controls between risk groups using a log-binomial model with 95% Wald confidence intervals after adjustment. In a sensitivity analysis, risk ratios were estimated stratifying women according to their breast density into tertiles of percentage mammographic density. In an analysis restricted to breast cancers, women were categorized into cancers diagnosed at stage 1 or lower and at stage 2 or higher. Risk ratios were reported by comparing the two tumor stage groups in women at high risk with women below high risk at study-entry after multivariable adjustment.

Statistical analyses were performed using R 4.1.^30^ All tests were two-sided with a significance level of 0.05.

### Role of the funding source

The study was funded by the Swedish Research Council (2022–06148), the Swedish Breast Cancer Association, the Mayo Clinic Comprehensive Cancer Center/Cancer Research Karolinska Institutet Collaborative Cancer Research Program, and iCAD Medical, Nashua, NH. The funders had no role in the study design, analyses, interpretation of data, writing the manuscript, approval, or decision to publish the results.

## Results

### Study population

The study included 8551 women, 739 incident breast cancers and 7812 controls, who were screened between 2009 and 2020 at four European screening populations in Italy, Spain, and Germany, Table 1, Supplementary Table S1. At study-entry, the mean age was 57.8 ± 5.7 in cases and 57.1 ± 5.1 in controls across screening populations, Table 1. The corresponding mean absolute 2-year risk was 1.07 ± 0.85 in cases and 0.57 ± 0.48 in controls. Similar average risks were observed at each population in controls. Women were screened using digital mammography modalities from GE, FUJI, Hologic, and Siemens, Supplementary Table S2.

**Table 1:** Baseline characteristics of 8551 women at four screening populations in the European validation study.

Population	N	Age^a^	PFAI risk 2-year^a^
*All populations combined*			
Cases	739	57.8 (5.7)	1.07 (0.85)
Controls	7812	57.1 (5.1)	0.57 (0.48)
*RETomo*			
Cases	180	57.4 (6.9)	1.17 (0.94)
Controls	1899	58.7 (5.6)	0.58 (0.51)
*Hospital Del Mar*			
Cases	158	57.8 (5.3)	0.98 (0.85)
Controls	1550	58.8 (5.4)	0.58 (0.49)
*München Süd*			
Cases	232	58.0 (5.2)	1.00 (0.72)
Controls	3124	55.5 (4.2)	0.56 (0.46)
*Paderborn*			
Cases	169	57.8 (5.3)	1.16 (0.92)
Controls	1239	56.8 (4.8)	0.54 (0.50)

PFAI Risk – ProFound AI risk tool.

RETomo – Italy, Hospital Del Mar–Spain, München Süd – Germany, Paderborn – Germany.

a Mean (SD).

### Breast cancers at follow-up

Breast cancers were diagnosed between 3 months and 2 years and 3 months in the study, Supplementary Fig. S1. Three of the four screening populations included non-screen detected breast cancers diagnosed between study-entry and the next scheduled screen. RETomo also included women aged 45–49 who had annual screens. The mean age at breast cancer diagnosis was 59.8 ± 5.8 across populations, Supplementary Table S3. A similar mean age at diagnosis was observed at each population. Breast cancers at stage 2 or higher were diagnosed in 25% of the cases and, similar percentages of later stage breast cancers were seen across populations in the range of 20–26%.

### Discriminatory performance overall and by screening population

The overall adjusted discriminatory performance across populations was aAUC 0.72 (95% CI 0.70–0.75) after 2 years of follow-up. aAUCs ranged from 0.71 (95% CI 0.67–0.74) to 0.74 (95% CI 0.69–0.78) after adjustment, Table 2. Similar performances were observed for women in the RETomo trial where women after risk assessment on a digital mammography modality were assessed for breast cancer at the next screen using a combination of digital mammography and digital breast tomosynthesis in one arm and digital mammography only in the second arm.

**Table 2:** Discriminatory performance of the PFAI Risk model by study population. AUCs were adjusted for mammography vendor, year of mammogram, and age at study-entry (aAUC).

Study population	N cases/controls	aAUC (95% CI)
RETomo, arm 1	89/984	0.73 (0.67–0.79)
RETomo, arm 2	91/915	0.73 (0.68–0.79)
Hospital Del Mar	158/1550	0.71 (0.67–0.75)
München Süd	232/3124	0.71 (0.67–0.74)
Paderborn	169/1239	0.74 (0.69–0.78)
All studies combined	739/7812	0.72 (0.70–0.75)

The aAUC for studies combined were additionally adjusted for study population.

Arm 1 – Risk measured on DM mammograms at baseline in our study in the women who were examined using DM at study-entry in the RETomo trial.

Arm 2 – Risk measured on DM mammograms at baseline in our study in the women who were examined using DM + DBT at study-entry in the RETomo trial.

aAUC – Adjusted Area Under the receiver operating characteristic Curve. DM – full-field Digital Mammogram DBT – Digital Breast Tomosynthesis.

The adjusted ROC curve presented sensitivities and specificities across all possible operating points from using a low-risk cut-off to a high-risk cut-off for referring a woman to additional examinations after a negative screen, Supplementary Fig. S2.

### Risk classification using NICE and USPSTF guidelines

**Fig. 1:**
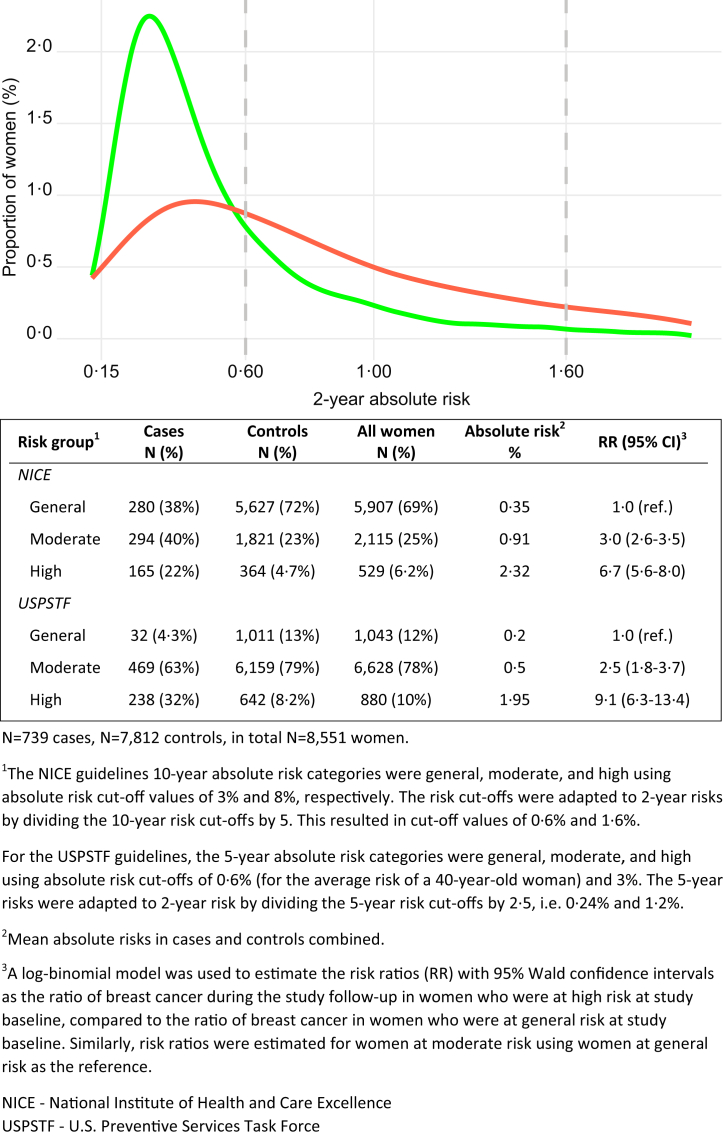
Frequency distribution of absolute 2-year risks at study-entry for developing breast cancer in cases (red) and controls (green) and, risk classification of women into high, moderate, and general risk using the NICE and USPSTF guidelines. Risk classification was additionally performed in women using USPSTF guidelines. Risk ratios were adjusted for study population, mammography vendor, year of mammogram, and age at study-entry.

Fig. 1 presents the frequency distribution of the absolute 2-year risks in the cases and controls for the four populations combined. Risk scale cut-offs were also presented for classifying women into risk categories using the NICE and USPSTF guidelines. Using the NICE guidelines thresholds for risk classification, 6.2% of the women in the study were classified as high risk at study-entry, 4.7% of the controls, and 22% of the women who were later diagnosed with breast cancer. Women classified as high-risk were at 6.7 (95% CI 5.6–8.0) times higher risk than women at general risk of breast cancer after adjustment for potential confounders. Using the USPSTF guidelines thresholds for risk classification, the corresponding numbers were 10%, 8.2%, 32%, and RR 9.1 (95% CI 6.3–13.4), respectively. Similar numbers were also observed per NICE and USPSTF guidelines in women in age ≥ 50, Supplementary Table S4.

Similar percentages of cases and controls at high risk were observed at each population with risk ratios between high-risk women and women at general risk ranging from 5.5 (95% CI 3.6–7.9) to 7.1 (95% CI 5.1–9.9) using NICE guidelines, Supplementary Table S5. Using the USPSTF guidelines, the percentage of controls that were at high risk at study-entry ranged from 6.5–9.8% and between 24 and 38% for cases, Supplementary Table S6.

### Risk stratification by mammographic density and by tumor stage

Table 3 presents risk ratios between high-risk women and women at general risk per NICE and USPSTF guidelines stratified by tertiles of mammographic density in order to analyze any influence that the risk model may have from mammographic density. Per NICE guidelines, women who were at high risk and were in the lowest density tertile had 5.8 (95% CI 3.8–8.6) times higher risk than women at general risk after adjustment. In comparison, women who were at high risk and were in the middle density tertile had a 6.7 (95% CI 4.9–9.2) times higher risk than the reference group. We observed similar numbers for the high-risk group in the highest density tertile, 6.1 (95% CI 4.7–7.9).

**Table 3:** Risk ratios with 95% Wald confidence intervals of the risk of breast cancer at study-entry per NICE and USPSTF guidelines stratified by tertiles of percent mammographic density from lowest (Q1) to highest (Q3).

Clinical guideline^a^	Q1, RR (95% CI)^b^	Q2, RR (95% CI)^b^	Q3, RR (95% CI)^b^
*NICE*^a^			
General	1 (ref.)	1 (ref.)	1 (ref.)
Moderate	3.6 (2.6–4.9)	3.4 (2.6–4.4)	2.2 (1.7–2.9)
High	5.8 (3.8–8.6)	6.7 (4.9–9.2)	6.1 (4.7–7.9)
*USPSTF*^a^			
General	1 (ref.)	1 (ref.)	1 (ref.)
Moderate	1.8 (1.1–3.3)	2.0 (1.1–4.0)	2.8 (1.6–5.3)
High	6.1 (3.2–12.2)	8.6 (4.6–17.6)	8.9 (5.0–17.5)

In each density strata the lowest risk group was used as the reference. Risk ratios (RR) were adjusted for study population, mammography vendor, year of mammogram, and age at study-entry.

NICE - National Institute of Health and Care Excellence. USPSTF - U.S. Preventive Services Task Force.

a The NICE guidelines 10-year absolute risk categories were general, moderate, and high using absolute risk cut-off values of 3% and 8%, respectively. The risk cut-offs were adapted to 2-year risks by dividing the 10-year risk cut-offs by 5. This resulted in cut-off values of 0.6% and 1.6%.For the USPSTF guidelines, the 5-year absolute risk categories were general, moderate, and high using absolute risk cut-offs 0.6% (for the average risk of a 40-year-old woman) and 3%. The 5-year risks were adapted to 2-year risk by dividing the 5-year risk cut-offs by 2.5, i.e. 0.24% and 1.2%.

b A log-binomial model was used to estimate the risk ratios with Wald 95% confidence intervals in tertiles of percent mammographic density using the fully automated STRATUS density tool defined by tertiles in controls.

Table 4 presents women diagnosed with stage 2 or higher breast cancer in comparison with breast cancers diagnosed at earlier stages. Breast cancers are further compared by their risk category at study-entry. Per NICE risk guidelines, breast cancers were 1.39 (95% CI 1.04–1.82) times more likely to be diagnosed at late stage than early stage during follow-up in women who were at high risk at study-entry compared with women who were below high risk at study-entry, after adjustments including time to diagnosis. The corresponding risk ratio using USPSTF guidelines was 1.33 (95% CI 1.01–1.74).

**Table 4:** Risk classification at study-entry per NICE and USPSTF guidelines stratified by women diagnosed with earlier stage and later stage breast cancer during study follow-up.

Clinical guideline^a^	Stage 1 or earlier, N (%)	Stage 2 or higher, N (%)	RR (95% CI)^b^
*NICE*			
General	211/533 (40%)	51/170 (30%)	1 (ref.)
Moderate	215/533 (40%)	69/170 (41%)	
High	107/533 (20%)	50/170 (29%)	1.39 (1.04–1.82)
*USPSTF*			
General	24/533 (4.5%)	7/170 (4.1%)	1 (ref.)
Moderate	350/533 (66%)	95/170 (56%)	
High	159/533 (30%)	68/170 (40%)	1.33 (1.01–1.74)

Women at high risk are compared with women not at high risk. Risk ratios were adjusted for participating study, mammography vendor, year of mammogram, age at study-entry, and time from mammogram to diagnosis.

Women with missing information on stage (N = 36) were excluded from the analysis.

NICE - National Institute of Health and Care. USPSTF - U.S. Preventive Services Task Force.

a The NICE guidelines 10-year absolute risk categories were general, moderate, and high using absolute risk cut-off values of 3% and 8%, respectively. The risk cut-offs were adapted to 2-year risks by dividing the 10-year risk cut-offs by 5. This resulted in cut-off values of 0.6% and 1.6%. For the USPSTF guidelines, the 5-year absolute risk categories were general, moderate, and high using absolute risk cut-offs 0.6% (for the average risk of a 40-year-old woman) and 3%. The 5-year risks were adapted to 2-year risk by dividing the 5-year risk cut-offs by 2.5, i.e. 0.24% and 1.2%.

b The risk exposure was dichotomized into high-risk and non-high risk (moderate and general risk) using the NICE guidelines. The non-high-risk group was used as the reference. The outcome was defined as stage 2 or higher breast cancers versus stage 1 or earlier breast cancers. A log-binomial model estimated the risk ratios (RR) with Wald 95% confidence intervals as the ratio of outcome in women who were at high risk at study baseline, compared to the ratio of outcome in women who were at non-high risk at study baseline. Similarly, the corresponding risk ratio was estimated using the USPSTF guidelines to define high-risk and non-high risk (i.e. moderate and general risk combined).

## Discussion

In an external validation in four European screening populations, we investigated the discriminatory performance and risk classification of an image-derived AI-based risk short-term model designed to identify women who are at high risk of breast cancer before or at the next screen after a negative screen. The AI-based risk model showed a similar discrimination to that of the original report, aAUC 0.72.^15^ Similar risk stratification performances were observed in women with dense and non-dense breasts. Late-stage breast cancers were more likely to be diagnosed in women at high risk than women at general or moderate risk.

Studies have shown that performing additional examinations using a more sensitive modality after a negative screen increases the detection of breast cancers in an unselected population and in women with dense breasts.^31^^,^^32^ By performing risk assessment beyond mammographic density to identify a subgroup of women that are more likely to benefit from additional examinations, screening outcomes may improve.

Modelling using deep learning has revived mammographic image analysis and resulted in the reporting of high predictive performances with a promise for using AI-based tools in the clinic.^13^^,^^33^ At the same time, the use of AI in clinical practice raises critical questions regarding the generalizability of the models. Models are developed in a fraction of the screening material where they potentially could be used. A recent report showed that when exchanging the data used to train and evaluate breast cancer screening models in the Digital Mammography Dialogue on Reverse Engineering Assessment and Methods (DREAM) Mammography Challenge to a more diverse screening population, substantially reduced predictive performances were observed.^34^ This stresses the need for evaluating a model in a diverse population and for its intended use before considering its clinical use.

The model used in our study was developed in a screening cohort in the Swedish screening setting.^15^^,^^23^ In the current study, we used what is referred to as Model 1, which includes mammographic features and age only. This model was originally reported to have a discriminatory performance (AUC 0.73 without age adjustment) similar to our current report.^15^ In our current study, we observed a small variability of discriminatory performances across populations of different European countries. The original and current reports show similar risk stratification performance when comparing women with high and general risk of breast cancer. The original and current report also show increased performance in later stage breast cancers compared with earlier stage breast cancers.

It should be underlined that the performance of an image-based AI-risk model could be influenced by ethnic differences and screening routines. In Europe with national screening programs, biennial screening is most often performed using digital mammography including double reading of mammograms, no supplemental screening, and a recall rate of 3–5%.^35^ In the U.S. screening setting with opportunistic screening, annual digital breast tomosynthesis screening is commonly performed with single reading of mammograms, additional supplemental screening may be performed, and recall rates of ∼10%.^36^ In consequence, in United States compared with Europe, cancers are diagnosed at an earlier stage, more in-situ tumors are diagnosed, and fewer interval cancers are diagnosed between the shorter screening intervals. The differences in screening settings could influence the risk model performance for several reasons. One reason is that a risk model could be trained in one screening setting with specific distributions of mammographic features associated with the screening setting, which could lead the model to be underspecified for another screening setting.^37^ Another reason is that cancer detection rates can vary 2–4-fold across screening units depending on the radiologists’ mammogram interpretation, which leads to differential outcome misclassification between screening populations.^36^ It could be that image-based risk models benefit from adapting to specific screening settings.^38^, ^39^, ^40^

The risk model in our study indicated a similar performance for capturing future diagnosed breast cancers in high-risk women with dense and non-dense breasts compared with women at a general risk of breast cancer. Women with non-dense breasts are more likely to develop more aggressive interval cancers.^6^ Women with dense breasts are more likely to have a tumor masked by dense tissue, which increases the risk of interval cancer and late stage breast cancer.^41^ Masking of a tumor by dense tissue is a classic radiologist challenge and, risk assessment using AI has the potential to improve screening outcomes. It could be that high-risk women with dense breasts are likely to benefit from a more sensitive modality following negative screening, while high-risk women with non-dense breasts are more likely to benefit from a shorter screening interval due to the increased risk of a fast-growing tumor. Therefore, a combination of density and risk assessment might be the way forward for risk stratification in population-based screening programs.

Clinical guidelines in the United States that recommend risk assessment are currently restricted to models that include a family history of breast cancer or breast density.^8^ Image-based risk models beyond density are currently not regulated in the United States or in Europe.^42^ A recent study indicated that an image-based model not only showed an overall higher discriminative performance compared to a clinical lifestyle/familial-based risk tool, but also higher discriminatory performances across subgroups of women by established risk factors of breast cancer and by breast cancer subtypes.^29^ Considering the reporting from several research groups on the performance of image-based risk models, such newer approaches for identifying women in need to supplemental screening and a shorter screening interval may be considered in updated screening guidelines.^39^^,^^43^, ^44^, ^45^ However, the risk-benefit balance of these models at an individual and societal level needs to be assessed before their clinical implementation for an individualized screening approach.^46^

This study has several limitations. We investigated model performance in a retrospective study at four screening populations performing biennial screening in age 45–69. We investigated the model performance in women with early and late stage of breast cancer, but we had no access to well-annotated data on interval cancers. We were also lacking interval cancer in one of our study populations. However, in a previous study we observed similar results in screen detected and interval cancers, which suggests that this lack of information may have little impact in our results.^15^ We had no access to family history and lifestyle risk factor data and therefore could not investigate the lifestyle/family history expanded version of the image-based risk model and compare to traditional lifestyle/familial risk models such as Tyrer-Cuzick and Gail.^47^^,^^48^ We also included a smaller proportion of women below age 50 with 1-year screening interval to reflect the screening routine in the Italian population. When restricting the overall study population to the most commonly used screening age of 50 and above, we observed similar percentages of women classified into the different risk categories using the NICE or USPSTF guidelines.

In conclusion, the image-derived AI-based risk model showed a generalized performance for identifying and classifying the risk of breast cancer in four European screening populations. The model predicts clinically relevant stage 2 and higher breast cancers in women who are at high risk of breast cancer before or at the next screen and are sent at home with a negative mammogram. An image-derived AI model is feasible for personalized breast cancer screening to improve the screening outcomes.

## Contributors

ME conceptualized and designed the study. AG, XC, AN, PP, SHK, PGR contributed to administrative, technical, or logistic support. MR, AG, XC, AN, PP, SHK, PGR contributed to collection and assembly of data. ME, AG, XC, AN, PP, SHK, PGR contributed to analysis and interpretation of the data. ME and PGR contributed to statistical expertise. ME drafted the article. ME, MR, AG, XC, AN, PP, SHK, PGR contributed to critical revision of the article for important intellectual content. All authors had full access to all data and had final responsibility for the decision to submit for publication.

## Data sharing statement

The data supporting the findings of this study fall under GDPR legislation and are available from authors upon reasonable request. The study protocol is available as an Appendix.

## Declaration of interests

ME has a patent on system and method for assessing breast cancer risk using imagery with a license to iCAD, Nashua NH.
