# Supplementary material for: European validation of an image-derived AI-based short-term risk model for individualized breast cancer screening—a nested case-control study
Source: Lancet Reg Health Eur. 2023 Dec 6;37:100798. doi: 10.1016/j.lanepe.2023.100798 (PMC10866984; doi:10.1016/j.lanepe.2023.100798)
Supplement: Supplementary Methods S1–S3, Tables S1–S6, and Figs. S1 and S2 [file mmc1.docx]

**Supplemental Material for**

Mikael Eriksson et al. European Validation of an Image-derived AI-based Short-term Risk Model for
Individualized Breast Cancer Screening – a Nested Case-Control Study

Contents

[Supplementary Methods 2](#_Toc149129639)

[Supplementary Method S1. Detailed description of four screening populations 2](#_Toc149129640)

[Supplementary Method S2. Description of mammographic risk factors in the risk model 5](#_Toc149129641)

[Supplementary Method S3. Description of adjusted AUC (aAUC) 7](#_Toc149129642)

[Supplementary Tables 9](#_Toc149129643)

[Supplementary Table S1. Overview of four screening cohorts 9](#_Toc149129644)

[Supplementary Table S2. Mammography machines 10](#_Toc149129645)

[Supplementary Table S3. Tumor characteristics 11](#_Toc149129646)

[Supplementary Table S4. Frequency distribution of absolute risks in women age ≥50 12](#_Toc149129647)

[Supplementary Table S5. Risk classification per NICE guidelines 13](#_Toc149129648)

[Supplementary Table S6. Risk classification per USPSTF guidelines 14](#_Toc149129649)

[Supplementary Figures 15](#_Toc149129650)

[Supplementary Figure S1. Days from screening to breast cancer diagnosis 15](#_Toc149129651)

[Supplementary Figure S2. ROC curve 16](#_Toc149129652)

# Supplementary Methods

## Supplementary Method S1. Detailed description of four screening populations

*RETomo*

Women aged 45 to 69 attending screening from March 2014 to August 2017 in one of the three screening clinics in Reggio Emilia with a screening history of at least one mammogram were eligible for the RETomo trial ^1^. Exclusion criteria were previous breast cancer, being included in or eligible for a hereditary breast cancer surveillance program ^2^ , pregnancy, previous Digital Breast Tomosynthesis (DBT) exam, very large breasts (which could require more than one exposure for each projection), augmentation prostheses, or language barriers. Women aged 45 to 49 were screened annually, while women aged 50 to 69 were screened biennially. Women in the trial are actively followed up to the age of 74. The participation rate in the trial was 94%. Women were randomized into two arms, one arm where women were screened with digital mammography (DM) and one arm where women were screened with DM and DBT combined. In both arms, two experienced radiologists independently read the images; in the event of disagreement, arbitration by a third reader was conclusive, as it is for routine screening.

In our current study, the women who participated in the a trial comparing the two arms of DM and DM plus DBT ^3^, were eligible for selection in accordance with our protocol for the nested case-control study, **Appendix A**. Mammograms and age were collected together with breast cancer status and tumor characteristics from medical records and cancer register.

*Hospital Del Mar*

Women from age 50 to 69 who attended screening between 2013-2020 in the breast cancer screening program at Hospital Del Mar were eligible for the study. Women with a personal history of breast cancer were excluded from the study. The breast cancer screening program at Hospital Del Mar is a publicly funded program that started in 1997, and follows the recommendations of the European Guidelines ^4^. Women in the age range 50 to 69 years are invited every two years by post letters to attend an appointment for a two-view screening mammography (craniocaudal, and mediolateral oblique) of each breast. Over 16,000 women are screened annually at Hospital Del Mar. The program has an average participation rate of 67% of invited women, and a re-attendance rate of 91·2%. Screening mammograms are read by certified breast radiologists by double-blind reading with arbitration by a third radiologist in the event of disagreement. Women with abnormal mammographic findings are recalled for further assessment to rule out malignancy. Those with negative assessment are referred back to regular screening invitation at 2 years. The center prospectively registers information on patient-related factors, screening mammography examinations, recall, further assessments, and breast cancer diagnoses.

In our current study, the cases and controls from women screened using Fuji full-field digital mammography (FFDM) were eligible for selection in accordance with our protocol for the nested case-control study, **Appendix A**. Mammograms and age were collected together with breast cancer status and tumor characteristics from medical records and cancer register.

*München Süd*

Women from age 50-69 who attended screening between 2015-2017 at the Münich screening unit in Bavaria, Germany, were eligible for the study. Women with a personal history of breast cancer were excluded from the study (by law). The München screening unit complies with the German biennial screening program. Breast cancer registration in Bavaria and underlies Bavarian law. The general German screening program addresses asymptomatic women aged 50-69 and, women with high-risk family history participate in familial high-risk program. Two radiologists are reading the mammograms independently of each other and, in the event of a disagreement, arbitration is performed by the lead radiologist and both readers.

In our current study, the women in the general screening program were eligible for selection in accordance with our protocol for the nested case-control study, **Appendix A**. Mammograms and age were collected together with breast cancer status and tumor characteristics from medical records.

*Paderborn*

Women aged 50-69 who attended screening between 2009 and 2016 at the two available sites in Paderborn were eligible for the study. Women who had a personal history of breast cancer were excluded from the study (by law). The mammography screening in Paderborn is part of the German biennial screening program ^5^. The screening unit addresses asymptomatic women. Women at known high risk participate in familial high-risk programs. Screening outcomes are registered in the MaSc system. Two radiologists were reading the mammograms independently of each other and, consensus conference was performed by all readers of the screening unit in case of disagreement. Breast cancer status on symptomatic breast cancers diagnosed between regular screens are not registered in MaSc and these cancers were not available from the regional cancer register, State Cancer Registry North Rhine Westphalia in Bochum in Germany, for this study.

In our current study, the women in the Paderborn screening population were eligible for selection in accordance with our protocol for the nested case-control study, **Appendix A**. Mammograms and age were collected together with breast cancer status and tumor characteristics from medical records and cancer register.

## Supplementary Method S2. Description of mammographic risk factors in the risk model

We present a detailed description of the mammographic features of the risk model as previously published ^6,7^. Mammographic density was measured using the STRATUS machine learning based tool. STRATUS starts by reading the DICOM formatted full-field digital and analogue mammogram and for the digital images also the mammography machine acquisition parameters. STRATUS thereafter quality checks the mammograms by inspecting image size, availability of patient identifier, mammogram date and time, mammogram view position, description of the mammogram series and study, and breast implants. Images are further normalized to 200-micron pixel size. STRATUS flips the images, so the breast chest wall always appears to the left-hand side in the mammogram, and inverts the LookUp-Table if the mammogram appears as a negative (i.e. when the pixel intensity representation is reversed).

STRATUS marks the breast area using threshold methods Intermodes, Triangle, or Means depending on the quality of the image. The next step identifies textures of the image in a cycle of 15 threshold methods (Otsu, RenyiEntropy, Huang, Intermodes, IsoData, Li, MaxEntropy, Mean, MinError, Minimum, Moments, Percentile, Shanbhag, Triangle, Yen) and one edge tracing method (Skeletonize). Twenty features (area, min, mean, max, std, modal, centroid, center, perimeter, bounding, fit, shape, integrated, median, skewness, kurtosis, limit, round, solidity, area_fraction) are measured for each cycle on the whole segmented breast area and also stratified by the size of textures in the breast area. Mammography machine acquisition parameters are added as extra information to the measures of image features including the x-ray exposure used (kilo volt and tube current) and the compression force and the thickness of the breast during compression. All features are measured for each threshold method and compiled into one row with up to 1,027 variables per digital mammogram. The variables are ordered starting with a variable holding the value for the first threshold method and first feature, i.e. OtsuArea. The second variable is OtsuMin and the last threshold-feature variable is YenSkeletonize.

Based on the extracted mammographic features, mammographic density is estimated using scaled principal component analysis (PCA). The original image reference measures (percent density, dense area, breast area) are transformed to a distribution close to normal form using square root transform. One hundred loops of model fits with ten-folded cross-validation were used in the training to calculate the mean lambda that is used to penalize the estimates of the mammographic features for the final density measurement model. This procedure is done for percent density, dense area, and breast area. The outcome measures are then back transformed to original density distribution by powering the values with two.

Microcalcifications and mass lesions were analyzed using an artificial intelligence neural network and, were considered indicative of high risk of breast cancer based on analysis of 60 regions in each projection (MLO and CC) of both breasts, i.e. in total 240 regions for the four views. Each subregion for each breast was assigned a risk probability ranging from 0 to 100% for microcalcifications and masses separately.

The first microcalcification score (high-risk) was continuous and defined as the highest probability measured in any of the subregions at risk of breast cancer by ordering the probabilities of microcalcifications from each region using the maximum probabilities of left and right breast views. The highest probability of a subregion indicating a mass at risk of breast cancer was calculated similarly by ordering the masses probabilities from each region using the maximum probabilities of left and right breast views.

The second microcalcification score (increased risk) was a dichotomous score based on the multiple subregions in each breast that had increased probability of microcalcifications in the breast. The 80^th^ percentile was used as the cut-off to indicate increased risk based on the underlying distribution from the 240 microcalcification subregion probabilities derived from left and right breasts of the breast cancer cases. The second score for masses was calculated similarly using the 80^th^ percentile of the underlying probabilities from the 240 masses subregion probabilities in the breast ^c^ancer cases.

The third microcalcification score (left-right risk asymmetry) was a continuous score that defined the difference in the occurrence of mammographic features in the left breast compared with the right breast in the mediolateral and cranio-caudal views. The score was calculated by first ordering the subregion probabilities for each breast and view. Variance was calculated for each of the 60 regions across left and right breasts in the mediolateral and craniocaudal views. The final score was the sum of the variances. The third score for left-right difference of masses was similarly calculated as the standard deviation of the probability score differences from each breast region of left and right breast in mediolateral and cranio-caudal views.

Age was extracted from headers in the mammogram.

Estimates of the risk factors used by the model is presented in below table as previously published ^7^.

|  | **Risk estimates Model 1 OR (95% CI)** | |
| --- | --- | --- |
| **Risk factor** | **Premenopausal** | **Postmenopausal** |
| Mammographic density, mean (per STD) | 1·49 (1·31,1·70) | 1·35 (1·24,1·47) |
| Microcalcifications, mean (per STD) | 1·88 (1·66,2·14) | 1·63 (1·50,1·78) |
| Masses, mean (per STD) | 2·03 (1·76,2·34) | 2·00 (1·83,2·19) |
| Left-right difference of mammographic density (per STD) | 1·11 (0·98,1·25) | 1·01 (0·92,1·10) |
| Left-right difference of microcalcification (per STD) | 1·46 (1·30,1·65) | 1·26 (1·17,1·36) |
| Left-right difference of masses (per STD) | 1·64 (1·44,1·87) | 1·79 (1·64,1·96) |

STD (standard deviation)
OR (95% CI) – odds ratios with 95% confidence intervals.
All factors in the model were mutually adjusted for each other and age.

## Supplementary Method S3. Description of adjusted AUC (aAUC)

We present a detailed description of adjusted AUCs as previously published ^8^. In breast cancer screening populations, the average age of women at the time of breast cancer diagnosis is commonly different than the general average age of women attending screening. Age is a strong risk factor for breast cancer. The predictive performance of a risk model could therefore be over or underestimated in a population where there is a systematic difference between the age in cases compared with the age in controls. Also, other factors such as systematic differences between screening sites and vendors could bias the estimation of the predictive performance if not adjusted for.

For this reason, we estimated the model performance using adjusted ROC curves and estimated AUCs based on covariate adjusted ROC curves.

The covariate-adjusted ROC (AROC) was defined as described by Janes et al. ^9^:

AROC(t) = $\int$ROC(t|x)dH_D_(x)

, where x is the adjusting covariate(s), t is operating point of the risk model, and H_D_(x) = Pr(X_D_ ≤ x) is the cumulative distribution function of the covariate vector for cases X_D_. The AROC curve is the average of covariate-specific ROC curve(s) and is weighted according to the distribution of the covariate(s) in cases.

The covariate-adjusted AUC (aAUC) was defined as described by Janes et al. as follows:

aAUC = $\int_{0}^{1}$AROC(t)dt = $\int_{0}^{1} \int$ROC(t|x)dH_D_(x)dt = $\int$AUC(x)dH_D_(x)

The estimations of the covariate-adjusted ROCs and AUCs in our study were performed as described by Carvalho & Rodriguez-Alvarez by using a Bayesian nonparametric method that is based on the combination of B-splines dependent Dirichlet process mixture models and the Bayesian bootstrap ^10^.

# Supplementary Tables

## Supplementary Table S1. Overview of four screening cohorts

Population representation and study design of four screening units in the European validation study.

| **Characteristic** | **RETomo** | **Hospital Del Mar** | **München Süd** | **Paderborn** |
| --- | --- | --- | --- | --- |
| Population representation | Screening unit | Screening unit | Screening unit | Screening unit |
| Screening years | 2014-2017 | 2013-2020 | 2015-2017 | 2009-2016 |
| Screening age | 45-67^1^ | 50-69 | 50-69 | 50-69 |
| Recall rate^2^ | 3·8% (both arms) | 5·2% | 4·5% | 2·5% |
| Detection rate^2^ | 4·5/1,000 (DM), 7·6/1,000 (DM+DBT) | 5·1/1,000 | 6·7/1,000 | 5/1,000 |
| Supplemental screening | None | None | None | None |
| Study design | RCT, nested case-control | Nested case-control | Nested case-control | Nested case-control |
| Screening interval | 1-yr: age <=50 2-yr: age>50 | 2-year | 2-year | 2-year |
| Screening modality | DM, DM+DBT^3^ | DM | DM | DM |
| Modality vendor | GE | Fuji | Hologic, Siemens | GE, Siemens |

^1^The inclusion of women in RETomo was in age 45-67, but women are actively followed-up till age 74.

^2^Average rates % and per 1,000 screens as reported from the screening units during the study period.

^3^Risk was assessed on DMs only in the RCT. Cancers that were detected as part of the RCT study were detected using DM in the standard screening arm and using DM+DBT in the intervention arm.

DM – Full-field Digital Mammogram
DBT – Digital Breast Tomosynthesis
RCT – Randomized Controlled Trial

## Supplementary Table S2. Mammography machines

Mammograms in the study by mammography machine vendor and case-control status.

| **Mammography vendor** | **All combined, N (%)^1^** | **Cases, N (%)^1^** | **Controls, N (%)^1^** |
| --- | --- | --- | --- |
| *Study populations combined* |  |  |  |
| FUJI | 1,708 / 8,551 (20%) | 158 / 739 (21%) | 1,550 / 7,812 (20%) |
| GE | 3,461 / 8,551 (40%) | 314 / 739 (42%) | 3,147 / 7,812 (40%) |
| Hologic | 1,274 / 8,551 (15%) | 65 / 739 (8·8%) | 1,209 / 7,812 (15%) |
| Siemens | 2,108 / 8,551 (25%) | 202 / 739 (27%) | 1,906 / 7,812 (24%) |
| *Individual screening populations* |  |  |  |
| RETomo (GE) | 2,079 / 8,551 (24%) | 180 / 739 (24%) | 1,899 / 7,812 (24%) |
| Hospital Del Mar (FUJI) | 1,708 / 8,551 (20%) | 158 / 739 (21%) | 1,550 / 7,812 (20%) |
| München Süd (Siemens) | 2,070 / 8,551 (24%) | 167 / 739 (23%) | 1,903 / 7,812 (24%) |
| München Süd (Hologic) | 1,274 / 8,551 (15%) | 65 / 739 (8·8%) | 1,209 / 7,812 (15%) |
| Paderborn (GE) | 1,370 / 8,551 (16%) | 134 / 739 (18%) | 1,236 / 7,812 (16%) |
| Paderborn (Siemens) | 38 / 8,551 (0·4%) | 35 / 739 (4·7%) | 3 / 7,812 (<0·1%) |
| ^1^n / N (%) | | |  |

## Supplementary Table S3. Tumor characteristics

Tumor characteristics and age at time of diagnosis.

|  | **All combined** | **RETomo** | **Hospital Del Mar** | **München Süd** | **Paderborn** |
| --- | --- | --- | --- | --- | --- |
| **Characteristic** | **N = 739^1^** | **N = 180^1^** | **N = 158^1^** | **N = 232^1^** | **N = 169^1^** |
| Age at diagnosis | 59·8 (5·8) | 59·0 (7·2) | 60·0 (5·3) | 59·8 (5·1) | 60·1 (5·6) |
| Stage |  |  |  |  |  |
| 0 | 131 / 703 (19%) | 26 / 180 (14%) | 27 / 148 (18%) | 45 / 206 (22%) | 33 / 169 (20%) |
| 1 | 402 / 703 (57%) | 117 / 180 (65%) | 82 / 148 (55%) | 112 / 206 (54%) | 91 / 169 (54%) |
| 2 | 152 / 703 (22%) | 29 / 180 (16%) | 36 / 148 (24%) | 49 / 206 (24%) | 38 / 169 (22%) |
| 3 | 14 / 703 (2·0%) | 8 / 180 (4·4%) | 2 / 148 (1·4%) | 0 / 206 (0%) | 4 / 169 (2·4%) |
| 4 | 4 / 703 (0·6%) | 0 / 180 (0%) | 1 / 148 (0·7%) | 0 / 206 (%) | 3 / 169 (1·8%) |
| (Missing) | 36 | 0 | 10 | 26 | 0 |
| ^1^Mean (SD); n / N (%) | | |  |  |  |

## Supplementary Table S4. Frequency distribution of absolute risks in women age ≥50

Frequency distribution of absolute 2-year risks at study-entry for developing breast cancer in cases (red) and controls (green) and, risk classification of women into high, moderate, and general risk using the NICE and USPSTF guidelines for women age ≥50. Risk ratios were adjusted for study population, mammography vendor, year of mammogram, and age at study-entry.

| \| **Risk group^1^** \| **Cases**  **N (%)** \| **Controls**  **N (%)** \| **All women**  **N (%)** \| **Absolute risk^2^, %** \| **RR (95% CI)^3^** \| \| --- \| --- \| --- \| --- \| --- \| --- \| \| *NICE* \|  \|  \|  \|  \|  \| \| General \| 259 (37%) \| 5,599 (72%) \| 5,858 (69%) \| 0·35 \| 1·0 (ref.) \| \| Moderate \| 283 (40%) \| 1,814 (23%) \| 2,097 (25%) \| 0·91 \| 3·0 (2·6-3·6) \| \| High \| 159 (23%) \| 364 (4·7%) \| 523 (6·2%) \| 2·32 \| 6·6 (5·5-7·9) \| \| *USPSTF* \| \|  \|  \|  \|  \| \| General \| 25 (3·6%) \| 1,002 (13%) \| 1,027 (12%) \| 0·2 \| 1·0 (ref.) \| \| Moderate \| 448 (64%) \| 6,133 (79%) \| 6,581 (78%) \| 0·5 \| 2·7 (1·9-4·2) \| \| High \| 228 (33%) \| 642 (8·3%) \| 870 (10%) \| 1·95 \| 9·4 (6·3-14·8) \| |
| --- | --- | --- | --- | --- | --- | --- | --- | --- | --- | --- | --- | --- | --- | --- | --- | --- | --- | --- | --- | --- | --- | --- | --- | --- | --- | --- | --- | --- | --- | --- | --- | --- | --- | --- | --- | --- | --- | --- | --- | --- | --- | --- | --- | --- | --- | --- | --- | --- | --- | --- | --- | --- | --- | --- |

N=701 cases, N=7,777 controls, in total N=8,478 women.

^1^The NICE guidelines 10-year absolute risk categories were general, moderate, and high using absolute risk cut-off values of 3% and 8%, respectively. The risk cut-offs were adapted to 2-year risks by dividing the 10-year risk cut-offs by 5. This resulted in cut-off values of 0·6% and 1·6%.

For the USPSTF guidelines, the 5-year absolute risk categories were general, moderate, and high using absolute risk cut-offs of 0·6% (for the average risk of a 40-year-old woman) and 3%. The 5-year risks were adapted to 2-year risk by dividing the 5-year risk cut-offs by 2·5, i.e. 0·24% and 1·2%.

^2^Mean absolute risks in cases and controls combined.

^3^A log-binomial model was used to estimate the risk ratios (RR) with 95% Wald confidence intervals as the ratio of breast cancer during the study follow-up in women who were at high risk at study baseline, compared to the ratio of breast cancer in women who were at general risk at study baseline. Similarly, risk ratios were estimated for women at moderate risk using women at general risk as the reference.

NICE - National Institute of Health and Care Excellence
USPSTF - U.S. Preventive Services Task Force

## Supplementary Table S5. Risk classification per NICE guidelines

Risk classification of women by study population into high, moderate, and general risk of breast cancer was performed using the NICE guidelines. Risk ratios were adjusted for mammography vendor, year of mammogram, and age at study-entry.

| **Study population and risk group^1^** | **Cases, N (%)** | **Controls, N (%)** | **Absolute risk^2^, %** | **RR (95% CI)^3^** |
| --- | --- | --- | --- | --- |
| *RETomo* |  |  |  |  |
| General | 66 / 180 (37%) | 1,320 / 1,899 (70%) | 0·33 | 1·0 (ref.) |
| Moderate | 64 / 180 (36%) | 477 / 1,899 (25%) | 0·92 | 2·8 (2·0-3·8) |
| High | 50 / 180 (28%) | 102 / 1,899 (5·4%) | 2·23 | 6·9 (5·0-9·6) |
| *Hospital Del Mar* |  |  |  |  |
| General | 76 / 158 (48%) | 1,147 / 1,550 (74%) | 0·39 | 1·0 (ref.) |
| Moderate | 57 / 158 (36%) | 348 / 1,550 (22%) | 0·88 | 2·5 (1·8-3·4) |
| High | 25 / 158 (16%) | 55 / 1,550 (3·5%) | 2·58 | 5·5 (3·6-7·9) |
| *München Süd* |  |  |  |  |
| General | 78 / 232 (34%) | 2,244 / 3,124 (72%) | 0·35 | 1·0 (ref.) |
| Moderate | 111 / 232 (48%) | 729 / 3,124 (23%) | 0·89 | 3·4 (2·6-4·6) |
| High | 43 / 232 (19%) | 151 / 3,124 (4·8%) | 2·15 | 5·6 (3·9-8·0) |
| *Paderborn* |  |  |  |  |
| General | 60 / 169 (36%) | 916 / 1,239 (72%) | 0·32 | 1·0 (ref.) |
| Moderate | 62 / 169 (37%) | 267 / 1,239 (22%) | 0·91 | 3·1 (2·2-4·3) |
| High | 47 / 169 (28%) | 56 / 1,239 (4·5%) | 2·33 | 7·1 (5·1-9·9) |

^1^The NICE guidelines 10-year absolute risk categories were general, moderate, and high using absolute risk cut-off values of 3% and 8%, respectively. The risk cut-offs were adapted to 2-year risks by dividing the 10-year risk cut-offs by 5. This resulted in cut-off values of 0·6% and 1·6%.

^2^Mean absolute 2-year risks in the cases and controls combined.

^3^A log-binomial model was used to estimate the risk ratios (RR) with Wald 95% confidence intervals as the ratio of breast cancer during the study follow-up in women who were at high risk at baseline, compared to the ratio of breast cancer in women who were at general risk at study baseline. Similarly, risk ratios were estimated for women at moderate risk using women at general risk as the reference.

NICE - National Institute of Health and Care Excellence

## Supplementary Table S6. Risk classification per USPSTF guidelines

Risk classification of women by study population into high, moderate, and general risk of breast cancer using USPSTF guidelines. The breast cancer cases in the general-risk group were too few to estimate risk ratio and compare across populations.

| **Study population and risk group^1^** | **Cases, N (%)** | **Controls, N (%)** | **Absolute risk^2^, %** |
| --- | --- | --- | --- |
| *RETomo* |  |  |  |
| General | 15 / 180 (8·3%) | 359 / 1,899 (19%) | 0·19 |
| Moderate | 96 / 180 (53%) | 1,354 / 1,899 (71%) | 0·51 |
| High | 69 / 180 (38%) | 186 / 1,899 (9·8%) | 1·85 |
| *Hospital Del Mar* |  |  |  |
| General | 0 / 158 (0%) | 82 / 1,550 (5·3%) | 0·23 |
| Moderate | 120 / 158 (76%) | 1,368 / 1,550 (88%) | 0·49 |
| High | 38 / 158 (24%) | 100 / 1,550 (6·5%) | 2·05 |
| *München Süd* |  |  |  |
| General | 8 / 232 (3·4%) | 319 / 3,124 (10%) | 0·20 |
| Moderate | 156 / 232 (67%) | 2,550 / 3,124 (82%) | 0·48 |
| High | 68 / 232 (29%) | 255 / 3,124 (8·2%) | 1·84 |
| *Paderborn* |  |  |  |
| General | 9 / 169 (5·3%) | 251 / 1,239 (20%) | 0·19 |
| Moderate | 97 / 169 (57%) | 887 / 1,239 (72%) | 0·48 |
| High | 63 / 169 (37%) | 101 / 1,239 (8·2%) | 1·90 |

^1^The USPSTF guidelines for 5-year absolute risk categories were general, moderate, and high using absolute risk cut-offs of 0·6% (for the average risk of a 40-year-old woman) and 3%. The 5-year risks were adapted to 2-year risk by dividing the 5-year risk cut-offs by 2·5, i.e. 0·24% and 1·2%.

^2^Mean absolute 2-year risks in the cases and controls combined.

USPSTF - U.S. Preventive Services Task Force

# Supplementary Figures

## Supplementary Figure S1. Days from screening to breast cancer diagnosis

Number of days from screening mammogram at study-entry to diagnosis of breast cancer.

| **RETomo**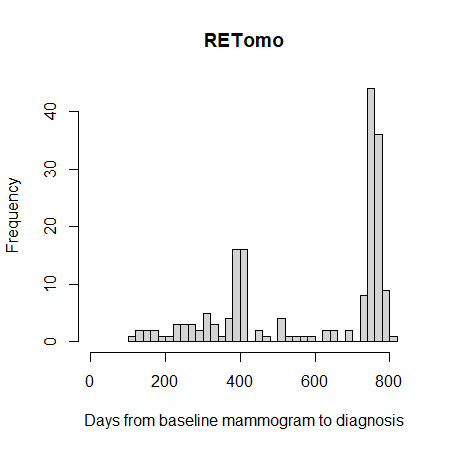 | **Hospital Del Mar**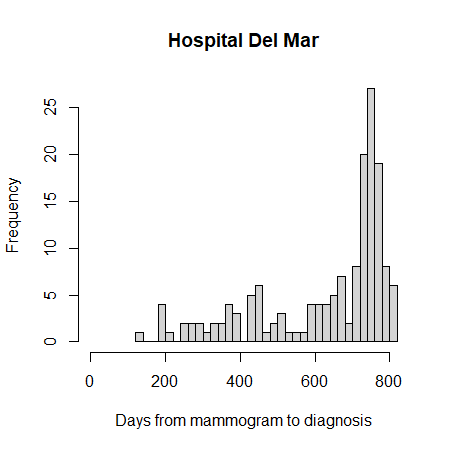 |
| --- | --- |
| **München Süd**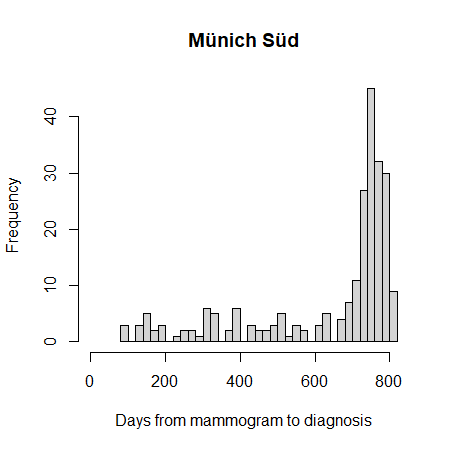 | **Paderborn**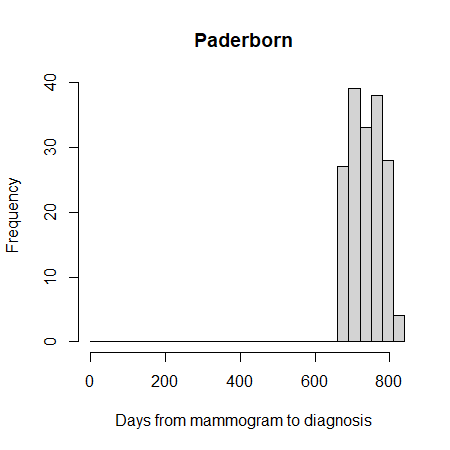 |

|  |  |
| --- | --- |
|  |  |
|  |  |

## Supplementary Figure S2. ROC curve

Receiver operating characteristic (ROC) curve with 95% confidence intervals for study populations combined after adjustment for study population, mammography vendor, year of mammogram, and age at study-entry. The adjusted ROC shows risk model sensitivities and specificities at different operating points of the risk model presented from low-risk operating points (upper right-hand side) to high-risk operating points (lower left-hand side).

**References**

1. Pattacini P, Nitrosi A, Giorgi Rossi P, et al. Digital mammography versus digital mammography plus tomosynthesis for breast cancer screening: the Reggio Emilia tomosynthesis randomized trial. *Radiology* 2018; **288**(2): 375-85.

2. Cortesi L, Baldassarri B, Ferretti S, et al. A regional population‐based hereditary breast cancer screening tool in Italy: First 5‐year results. *Cancer Medicine* 2020; **9**(7): 2579-89.

3. Pattacini P, Nitrosi A, Giorgi Rossi P, et al. A randomized trial comparing breast cancer incidence and interval cancers after tomosynthesis plus mammography versus mammography alone. *Radiology* 2022; **303**(2): 256-66.

4. European Commission Initiative on Breast Cancer Contributor Group. European Guidelines on Breast Cancer Screening and Diagnosis. Available online: <https://healthcare-quality.jrc.ec.europa.eu/european-breast-cancer-guidelines> (accessed July 2023).

5. Katalinic A, Eisemann N, Kraywinkel K, Noftz MR, Hübner J. Breast cancer incidence and mortality before and after implementation of the German mammography screening program. *International journal of cancer* 2020; **147**(3): 709-18.

6. Eriksson M, Li J, Leifland K, Czene K, Hall P. A comprehensive tool for measuring mammographic density changes over time. *Breast Cancer Res Treat* 2018; **169**(2): 371-9.

7. Eriksson M, Czene K, Strand F, et al. Identification of women at high risk of breast cancer who need supplemental screening. *Radiology* 2020; **297**(2): 327-33.

8. Eriksson M, Czene K, Vachon C, Conant EF, Hall P. Long-Term Performance of an Image-Based Short-Term Risk Model for Breast Cancer. *Journal of Clinical Oncology*; **0**(0): JCO.22.01564.

9. Janes H, Pepe MS. Adjusting for covariate effects on classification accuracy using the covariate-adjusted receiver operating characteristic curve. *Biometrika* 2009; **96**(2): 371-82.

10. de Carvalho VI, Rodriguez-Alvarez MX. Bayesian nonparametric inference for the covariate-adjusted ROC curve. *arXiv preprint arXiv:180600473* 2018.
