# Supplementary material for: European validation of an image-derived AI-based short-term risk model for individualized breast cancer screening—a nested case-control study
Source: Lancet Reg Health Eur. 2023 Dec 6;37:100798. doi: 10.1016/j.lanepe.2023.100798 (PMC10866984; doi:10.1016/j.lanepe.2023.100798)
Supplement: Supplementary Material [file mmc2.pdf]

Short-Term Image-Based Prediction of Breast Cancer Risk

Title	Short-Term Image-Based Prediction of Breast Cancer Risk
Study Principal Investigator	Karolinska Institutet, Medical Epidemiology and Biostatistics<br>Nobels väg 12A, 171 65 Stockholm, Sweden
Funder	iCAD Inc. 98 Spit Brook Road, Suite 100, Nashua, NH 03062 USA
Protocol date	28 January 2021

TABLE OF CONTENTS

TABLE OF CONTENTS **2**

1 RATIONALE **3**

2 BACKGROUND **3**

3 PROTOCOL SYNOPSIS **5**

3.1 OBJECTIVES AND ENDPOINTS 5
3.2 STUDY DESIGN, PROCEDURES AND PARTICIPANTS 6
3.2.1 Necessary Core Variables *6*
3.2.2 Mammograms *7*
3.2.3 Additional Variables, optional *7*

4 STUDY PROCEDURES **8**

4.1 SCREENING FOR ELIGIBILITY 8
4.2 DATA COLLECTION 8
4.3 DATA SAFETY AND MONITORING 8
4.4 DATA ACCESS, SHARING AND PUBLICATIONS 8

5 STATISTICAL ANALYSIS **9**

6 REFERENCES **10**

1 RATIONALE

Mammography screening reduces breast cancer mortality, but a proportion of breast cancers are missed and are detected at later stages or develop in between screening intervals. One possibility to detect breast cancers even earlier than done today would be to adopt a risk based screening approach. In a risk based screening approach women will have their screening tailored according to their risk of breast cancer and how difficult their mammograms are to interpret. To achieve such a goal, women have to be stratified on risk of breast cancer. ProFound AI® Risk is a risk prediction tool that identifies women that have a high likelihood to be diagnosed with breast cancer within 2 years.

ProFound AI Risk was developed using a Swedish mammography screening cohort and in collaboration between Karolinska Institutet and iCAD. The risk tool consists of three models. The first model uses image features derived from mammograms and age of the woman. The second model adds lifestyle risk factors and family history of breast cancer to the first model. The third model includes genetic determinants in addition to the image features, age, lifestyle factors, and familial risk factors.

The purpose of the project is to perform a retrospective validation of the ProFound AI Risk tool in women from different screening programs and populations.

2 BACKGROUND

Mammography screening was introduced in the 1970s and women in organized screening programs are invited based on age, commonly aged 50-69 for screening every 1-3 years. The overall aim of a screening program is to reduce breast cancer mortality through early detection. Several studies have indicated that mammography screening reduces breast cancer mortality by approximately 20% [1].

In most biennial screening programs ≈ 30% of all detected cancers are so called interval cancers, that is, they are detected between two screening intervals. There are three major reasons for an interval cancer to appear, it can either be missed, masked in dense tissue or fast growing and not visible at the prior screen. Interval cancers that are not masked or missed are known to have more aggressive tumor characteristics, and thereby worse prognosis, than screen detected cancers. Regardless of the reason for an interval cancer it has been suggested that these high risk women could benefit from an intensified screening intervention, such as shortening of screening intervals or adding supplemental screening methods. The concept of risk based screening builds on the ability to identify the individual risk of breast cancer and design the screening efforts according to individual risk and features of a mammogram.

The key factor for individualized screening is to assess the short-term risk of breast cancer, i.e. the risk of being diagnosed with a breast cancer before, or at the next mammographic screen. In other words, an ability to identify women that have a mammogram interpreted as negative, yet later, are diagnosed with an interval cancer. Such a risk model, ProFound AI Risk, has been developed at the Karolinska Institutet, Stockholm, in collaboration with iCAD [2].

The Karma Cohort [karmastudy.org] is a prospective screening cohort consisting of ≈70,000 women that has been followed for an average of 8 years [3]. The cohort was used for establishing the model and the model was further validated in two unique Swedish data sets.

The ProFound AI Risk consists of three models. Model 1 includes age and analyses of the mammograms through a machine learning technique. Mammographic features such as density, masses, microcalcifications and the asymmetry between left and right breasts are taken in to consideration. A further extension of the model (Model 2) includes lifestyle factors (use of exogenous hormones, alcohol and tobacco), menopausal status and family history of breast cancer in addition to image features. When adding genetic determinants to Model 2 we establish Model 3. The genetic determinants consists of 313 single nucleotide polymorphisms (SNP) identified by the Breast Cancer Association Consortium [4].

**Model**	**AUC**
1. Model 1; mammographic density, microcalcifications, masses, age	**0.73**
2. Model 2; Model 1 + lifestyle and familial risk factors	**0.74**
3. Model 3; Model 2 + genetic determinants	**0.77**
Validation dataset I (104 cancers, 9,745 healthy subjects), Model 1	**0.71**
Validation dataset II (613 cancers, 8,489 healthy subjects), Model 1	**0.73**
Tyrer-Cuzick + density	**0.62**
Gail + density	**0.61**

The discriminatory performances of the ProFound AI Risk are seen in the figure above. Model 1 reaches an AUC of 0.73 and improves to 0.77 in Model 3. Model 1 was validated in two independent datasets and reassuringly the same AUCs (0.71 and 0.73, respectively) as in the original dataset was seen.

There is no consensus on how risk based screening should be organized. Studies are ongoing, e.g. the European MyPebs [mypebs.eu] and the US Wisdom [wisdomstudy.org] trials but no results have been published. The risk models used in these trials are almost entirely based on genetics with no in depth analyses of patient-level mammographic features included. In contrast to the US, in Europe, no risk assessment is performed when women come for screening. In the US, the majority of states have made it mandatory to inform women about their mammographic breast density after a screening study has been performed. Risk models, such as Gail and Tyrer-Cuzick, are also used to identify women possibly in need of supplemental magnetic resonance imaging.

The Gail model was developed in the 1980s and includes age, family history of breast cancer, number of previous breast biopsies, age at menarche, and age at first birth [5]. The Tyrer-Cuzick risk model added factors such an extensive family history of breast cancer, BRCA mutation risk, low susceptible genetic breast cancer risk, menopausal status, and use of menopausal hormone therapy [6]. As can be seen in the table above, even after adding

mammographic density, the models are reaching an AUC of 0.62 (Tyrer-Cuzick) and 0.61 (Gail).

Even the most advanced of the ProFound AI Risk models, Model 3, do not include the large number of risk factors seen in the Gail and Tyrer-Cuzick models. The reason is that most lifestyle risk factors (e.g. age at first birth, breast feeding) are reflected in the mammographic density. Women who had their first child early in life and breast feed have a lower risk of breast cancer than nulliparous women and also have a lower density than age matched controls. This means that information on many risk factors could be substituted with mammographic features in predicting short-term risk of breast cancer. It also means that risk prediction using ProFound AI Risk is logistically, relatively easy. A woman that attends mammography screening could have her risk for breast cancer assessed as part of a screening visit without anything beyond consenting to the risk assessment.

The table below describes the risk stratification generated by the different ProFound AI Risk models in a Swedish screening population. As can be seen, there is no major difference between Model 1 and 2. Adding a polygenic risk score increase the ability to identify women at low risk of breast cancer as the low risk group increases from 4% to 27% comparing Model 1 and 3. The proportion of women at increased risk, moderate and high risk groups, stay the same, regardless of model.

**2-year risk of developing breast cancer**	**Model 1**	**Model 2**	**Model 3**
Low, <0.15%	4	6	27
General, 0.15-0.60%	60	58	48
Moderate, 0.60-1.60%	27	26	17
High, >1.60%	9	10	8

3 PROTOCOL SYNOPSIS

3.1 Objectives and Endpoints

- *The primary objective* is estimation of discriminatory performance and risk stratification of the risk tool in different screening programs and populations.
  - The primary endpoints are AUC and proportions of women in different risk categories in different screening programs and populations.
- *The secondary objective* is to identify and estimate *factors* that influence the primary objectives for overall breast cancer and tumor subtypes.
  - The secondary endpoint is the discriminatory performance and risk factor stratification in sensitivity analyses based on breast cancer incidence rates, competing mortality rates, race/ethnicities, background characteristics, personal screening history, image features, and risk factor prevalence.
- *The tertiary objective* is to identify and estimate image features that differ between digital mammography and tomosynthesis.

    - The tertiary endpoints are point estimates, standard errors, and distributions of image features in digital mammography and tomosynthesis and differences in point estimates and distributions.
- *The quaternary objective* is to compare the risk tool to existing risk tools based on discriminatory performance and risk stratification.
    - The quaternary endpoints are comparison based on point estimates, standard errors, and confidence intervals of AUCs and proportions of women in different risk categories using different risk models.

3.2 Study Design, Procedures and Participants

A meta-analysis will be performed based on cohort studies and case—controls studies that are sampled from mammography screening sites in Europe and the US. The aim is that the multi-center study should constitute 5 sites and the following prerequisites applies for each site:

3.2.1 Necessary Core Variables

The necessary core variables apply to women attending regular mammography screening according to the sites' screening program.

A detailed description of the variables are given in the document, **ProFound-AI-Risk_Data_Dictionary_v1.01.xlsx**. Access to the document is given by the Karolinska Institute study center at the Department of Medical Epidemiology and Biostatistics. Contact person; Mikael Eriksson. mikael.eriksson@ki.se.

A brief description is found below.

- Access to mammograms for at least 100 incident breast cancer cases.
    - *Mammograms prior to the imaging study at diagnosis are essential*
    - *Diagnostic mammograms could be included*
    - *Women with a previous history of breast cancer are not to be included*
- Access to mammograms for at least 500 healthy controls (i.e. not diagnosed with breast cancer during the study period).
    - *Cases and controls should be sampled from the same calendar period*
- Access to standard bilateral FFDM of MLO and CC views for presentation images
- Date of birth (year, month)
- Date of breast cancer diagnosis (year, month)
- Date of mammography (year, month)
- Date of last prior screening mammogram (year, month)
- National or regional breast cancer incidence rates
- National or regional mortality rates

3.2.2 Mammograms

Mammograms will either be collected at screening sites and transferred to the Karolinska Institute study center at the Department of Medical Epidemiology and Biostatistics, or analysis software and server will be provided to the screening unit for on-premise analysis.

- Any additional mammograms performed within minutes after the initial mammogram should also be included, e.g. tiled images or implant-displaced mammograms.
- Imaging characteristics from screening sites in Europe should be consistent with EUSOBI/EUSOMA Mammography Quality Standards in terms of positioning, compression, exposure level, dose, contrast, sharpness, noise, artifacts and the national guidelines and quality scheme of the screening unit.
- Imaging characteristics from screening sites in the US should be consistent with the Mammography Quality Standards Act (MQSA) in terms of positioning, compression, exposure level, dose, contrast, sharpness, noise and artifacts.

3.2.3 Additional Variables, optional

- Mammogram view position, laterality, mammographic density
- Description of lesions
    - Localization
    - Laterality
    - Type
        - Mass
        - Calcifications
        - Asymmetry
        - Architectural distortion
- Tumor characteristics
    - Tumor size, nodal status, metastasis, stage, grade, ER-status, PR-status, HER2-status, Ki67
- Risk factors
    - Date of interview (year, month)
    - Age
    - BMI
    - Alcohol use
    - Tobacco use
    - Menopausal hormone therapy use
    - Menopausal status

  - Family history of breast cancer
    - Breast cancer in mother, sisters, daughters
    - Age of diagnosis of the youngest relative with breast cancer
  - Genetic information (e.g., mutation screening of BRCA 1 and 2 and/or genotypes)
  - Ethnicity of mother and father by origin of country or race of the woman
- Risk tool information
  - Absolute risks from other risk tools, e.g. Tyrer-Cuzick, Gail.

4 STUDY PROCEDURES

4.1 Screening for Eligibility

Screening sites that express an interest in participating in the project will be screened for eligibility criteria. A prerequisite are access to core variables described in **3.2.1 Necessary Core Variables**.

The individual screening sites have to fully comply with the ethics requirements, research policies and procedures at their respective sites.

A Material Transfer Agreement has to be set up between the study site, Karolinska Institutet and iCAD to specify the policies and procedures agreed upon by the study site, Karolinska Institute and iCAD for transferring data, hardware and other study-related items between each other as needed to accomplish the study.

4.2 Data Collection

A retrospective data collection will be performed to gather data of the study participants as listed in the subsections of **3.2 Study Design, Procedures and Participants** and specified in the Data Dictionary (see **3.2.1 Necessary Core Variables**). In addition, breast cancer incidence and competing risk mortality rates will be collected from each screening site region and official national statistics.

4.3 Data Safety and Monitoring

Data should be collected at each site, anonymized and transferred, using encryption, to the Karolinska Institute study center at the Department of Medical Epidemiology and Biostatistics. The department has official documentation of data and safety monitoring plans available at www.ki.se/MEB/. The department plans comply with GDPR. [7]

4.4 Data Access, Sharing and Publications

A Steering Board for data access and publications will be formed consisting of each Principal Investigators, two representatives from Karolinska Institutet and two representatives from iCAD. The Steering Board grants access to data and decides on publication.

Data collected will only be used for the intended Objectives of the study. If additional projects are identified, data could only be used after first obtaining approval from the Principal Investigators at each site.

Principal Investigators are always granted access to their site specific data. Access to data from other sites will only be granted after approval from Principal Investigators of these sites.

The Karolinska Institutet has the responsibility to publish a paper based on the compiled data of the project. All Principal Investigators and three additional researchers from each sites will be listed as co-authors. Principal Investigators are thereafter encouraged to publish based on their own data.

5 STATISTICAL ANALYSIS

Baseline characteristics of the study participants will be summarized by standard descriptive summaries including means, standard deviations, confidence intervals and study group differences.

The primary objective of the project is estimation of discriminatory performance and risk stratification of the risk tool in different screening programs and populations. [8,9] The secondary endpoint is the discriminatory performance and risk factor stratification in sensitivity analyses based on breast cancer incidence rates, competing mortality rates, race/ethnicities, background characteristics, personal screening history, image features, and risk factor prevalence. [8,9] The tertiary and quaternary endpoints are point estimates, standard errors, and confidence intervals of AUCs and proportions of women in different risk categories using different risk models . [10,11]

6 REFERENCES

[1] B Lauby-Secretan, C Scoccianti, D Loomis et al.; Breast-cancer screening--viewpoint of the IARC Working Group; N Engl J Med. 2015 Jun 11;372(24):2353-8. doi: 10.1056/NEJMsr1504363.

[2] Mikael Eriksson, Kamila Czene, Fredrik Strand, Sophia Zackrisson, Peter Lindholm, Kristina Lång, Daniel Förnvik, Hanna Sartor, Nasim Mavaddat, Doug Easton, Per Hall. Identification of women at high risk of breast cancer who need supplemental screening. Radiology 2020, doi:0.1148/radiol.2020201620.

[3] Gabrielson M, Eriksson M, Hammarström M, Borgquist S, Leifland K, Czene K, Hall P. Cohort profile: The Karolinska Mammography Project for Risk Prediction of Breast Cancer (KARMA). Int J Epidemiol. 2017 Feb 9. doi: 10.1093/ije/dyw357. PMID: 28180256

[4] Mavaddat N, Michailidou K, Dennis J, Lush M, Fachal L, et al. Polygenic Risk Scores for Prediction of Breast Cancer and Breast Cancer Subtypes. Am J Hum Genet. 2019 Jan 3;104(1):21-34. doi: 10.1016/j.ajhg.2018.11.002. Epub 2018 Dec 13.

[5] MH Gail, LA Brinton, DP Byar, et al.; Projecting individualised probabilities of developing breast cancer for white females who are being examined annually; J Natl Cancer Inst 1989, 81:1879-1886.

[6] J Tyrer, SW Duffy and J Cuzick; A breast cancer prediction model incorporating familial and personal risk factors; Statist. Med. 2004; 23:1111–1130 (DOI: 10.1002/sim.1668).

[7] GDPR (EU) https://eur-lex.europa.eu/eli/reg/2016/679/oj

[8] G Schwarzer, JR Carpenter, G Rücker; Meta-analysis; Springer Nature ISBN 978-3-319-21416-0, 2015, Editon 1.

[9] JPT Higgins, S Green (editors); Cochrane Handbook for Systematic Reviews of Interventions Version 5.1.0 [updated March 2011]; The Cochrane Collaboration, 2011.

[10] ER DeLong, DM DeLong and DL Clarke-Pearson; Comparing the areas under two or more correlated receiver operating characteristic curves: a nonparametric approach; Biometrics 44, 837—845, 1988.

[11] NA Obuchowski, DK McClish; Sample size determination for diagnostic accuracy studies involving binormal ROC curve indices; Statistics in Medicine, 16, 1529--1542. DOI: 10.1002/(SICI)1097-0258(19970715)16:13<1529::AID-SIM565>3.0.CO;2-H, 1997.
